# Outcomes of retropupillary iris claw lens implantation in patients with intraocular lens dislocation and low (less than 1000 cells/mm^2^) corneal endothelial cell density

**DOI:** 10.1186/s12886-024-03621-4

**Published:** 2024-08-26

**Authors:** Seung Min Lee, Tae Young Kim, Hyun Goo Kang, Junwon Lee, Min Kim

**Affiliations:** 1 Department of Ophthalmology, Institute of Vision Research, Gangnam Severance Hospital, Yonsei University College of Medicine, 211 Eonjuro, Gangnam-gu, Seoul, 06273 Republic of Korea; 2 Department of Ophthalmology, Institute of Vision Research, Severance Hospital, Yonsei University College of Medicine, 134 Shinchon-dong, Seodaemun-gu, Seoul, Republic of Korea

**Keywords:** IOL dislocation, Retropupillary, Retropupillary Iris fixation, Endothelial cell density, Bullous Keratopathy

## Abstract

**Background:**

Posterior chamber intraocular lens (IOL) dislocation is a common complication of cataract surgery. Dislocated IOLs often require surgical intervention due to the potentially severe risks of leaving this condition untreated. If a patient with extremely low corneal endothelial cell density (ECD) presents with IOL dislocation, the surgeon faces a crucial dilemma of choosing the most optimal surgical treatment option. We sought to investigate the efficacy and safety of retropupillary iris claw intraocular lens (R-IOL) implantation in patients with IOL dislocation and extremely low (< 1000 cells/mm^2^) ECD.

**Methods:**

We retrospectively reviewed the medical records of nine patients (all men) whose pre-operative ECD was < 1000 cells/mm^2^ and who underwent R-IOL implantation due to intraocular subluxation or total dislocation into the vitreous cavity between 2014 and 2020. We evaluated corneal endothelial function and visual outcomes after surgery.

**Results:**

Nine patients were included in this study. The mean age at diagnosis was 64.89 ± 7.15 years (range 57–76 years), and the follow-up duration was 37.93 ± 23.72 months (range 18.07–89.07 months). No patients developed bullous keratopathy during follow-up. Compared to the initial ECD, corneal thickness (CT), coefficient variation of cell area (CV) and percentage of hexagonal cells (HEX), there was no statistically significant decrease in the ECD, CV, and HEX at last follow-up (*P* = 0.944, 0.778, 0.445, 0.443). There was significant improvement in the mean uncorrected distance visual acuity (UDVA) at the last follow-up (average 0.13 logMAR, 20/27 Snellen) compared to the pre-operative mean UDVA (average 1.09 logMAR, 20/250 Snellen) (*P* < 0.01).

**Conclusions:**

R-IOL implantation did not result in a statistically significant decline in corneal endothelial function in patients with preoperatively low ECD, and it significantly improved the mean UDVA postoperatively. R-IOL implantation appears to be a safe and effective treatment modality for intraocular lens dislocation in patients with low ECD (< 1000 cells/mm²); however, long-term follow-up studies are warranted to corroborate these findings.

## Background

Posterior chamber intraocular lens (IOL) dislocation is one of the most common late complications after cataract surgery [1]. Its incidence has been increasing over the past decades [1–5]. Because of the potential risk of decreased visual acuity, increased intraocular pressure, retinal detachment, and vitreous hemorrhage [2–6], dislocated IOLs often require surgical intervention. Secondary IOL scleral fixation has been a conventional treatment of choice; however, it requires advanced surgical skills and a longer operation time [7] and has the potential of causing suture-related complications, such as suture erosions, tilted lens, and choroidal and suprachoroidal hemorrhage [8]. To avoid such concerns, iris claw lenses with anterior or retropupillary implantations have been developed as alterative surgical options.

Many reports mention anterior chamber intraocular lens (ACIOL) implantation, wherein the IOL positions closer to the cornea have an increased risk of corneal endothelial cell damage and bullous keratopathy [9, 10]. Unlike that of ACIOL, the position of the retropupillary iris claw intraocular lens (R-IOL) is similar to the actual anatomical position, and significant endothelial cell loss after the surgery is not noted [11–14]. When the endothelial cell density (ECD) drops below 500 cells/mm^2^, dysfunction is likely, and corneal decompensation may occur, leading to chronic corneal edema and eventually bullous keratopathy [15]. Thus, if a patient with extremely low ECD presents with IOL dislocation, the surgeon faces a crucial dilemma of choosing the most optimal surgical treatment option. A treating surgeon must perform the most minimally invasive procedure to minimize ECD loss, while preserving the patient’s vision by successfully removing the IOL and fixating a secondary IOL. At times, surgeons might choose not to perform surgery at all if the risk/benefit ratio is deemed too high. In such circumstances, R-IOL could be considered as a valuable option, since its implantation is minimally invasive and required less surgical time compared to other suture fixations, thus minimizing potential endothelial cell damage in already compromised corneas.

There have been no reports on the efficacy and safety of R-IOLs in eyes with extremely low ECD. The aim of the present study was to describe the surgical outcomes of R-IOL (iris claw Artisan Aphakia Model 205, Ophtec BV, Groningen, The Netherlands) implantation in patients with IOL dislocation whose pre-operative ECD was < 1000 cells/mm^2^.

## Methods

This retrospective case series study was conducted at Gangnam Severance Hospital, which is affiliated with Yonsei University College of Medicine, Seoul, Korea. The study adhered to the tenets of the Declaration of Helsinki, and ethics approval was obtained from the Gangnam Severance Hospital Institutional Review Board (No. 3-2022-0225). The need for informed consent was waived because of the retrospective design of the study.

We reviewed the medical and operative records of all patients at our hospital who underwent R-IOL (iris claw Artisan Aphakia Model 205, Ophtec BV, Groningen, The Netherlands) implantation due to intraocular subluxation or total dislocation into the vitreous cavity between 2014 and 2020. The inclusion criteria for this study were as follows: (1) pre-operative ECD was < 1000 cells/mm^2^, (2) IOL was either subluxated or totally dislocated to the vitreous cavity, (3) R-IOL implantation and trans-pars plana vitrectomy were performed, and (4) follow-up duration was > 12 months. The following clinical characteristics were assessed for the study participants: (1) demographic data, (2) presence of symptoms, (3) cause of initial IOL insertion, (4) previous operation method for IOL insertion, (5) uncorrected distance visual acuity (UDVA) and/or best-corrected visual acuity (BCVA) both before and after surgery, (6) intraocular pressure (IOP) before and after the operation, and (7) corneal endothelial function data, which included pre- and post-operative measurements of ECD, corneal thickness (CT), efficient variation of cell area (CV), and the percentage of hexagonal cells (HEX). Vision was measured using a 6 m-Decimal chart, and responses were converted to Snellen and logarithm of the minimum angle of resolution (logMAR) values for statistical analysis. For non-numerical visual acuity, the following logMAR values were used for statistical calculation: 2.00 for count fingers (CF), 2.30 for hand movements (HM), 2.60 for light perception (LP), and 2.90 for no LP (NLP). IOP was measured using a noncontact tonometer. Biometric measurements were obtained using a ZEISS IOLMaster 500 (Carl Zeiss AG; Heidenheim, Germany), and IOL calculations were performed with the SRK/T formula using an A-constant of 116.9. ECD was calculated automatically using a specular microscope (CellChek XL, Konan Medical USA Inc., Irvine, CA, USA).

Under local or general anesthesia, a 25-gauge pars plana vitrectomy was performed in all patients. For local anesthesia, 1.5 ml of 2% lidocaine HCl and epinephrine (1:100,000) (Yuhan, Seoul, Korea) was injected into Tenon’s capsule. For mydriasis, two to three drops of 0.5% tropicamide and 0.5% phenylephrine (Tropherin^®^, Hanmi Pharmaceutical, Inc., Seoul, Korea) was applied. A 5.5-mm sclero-corneal tunnel was made at the 12 o’clock position, and two side incisions were made at the 2 o’clock and 10 o’clock positions. After freeing the dislocated IOL from the vitreous by partial vitrectomy, it was gently removed through the sclero-corneal tunnel. After filling the anterior chamber with a viscoelastic material (Viscoat, Alcon, Fort Worth, TX, USA), R-IOL (iris claw Artisan Aphakia Model 205) was inserted through a previously formed sclero-corneal tunnel. The lens haptics were enclavated to the posterior iris at the 3 o’clock and 9 o’clock positions. Miotic agent was not used before implanting the R-IOL. Inadvertent touch of the iris usually occurs during the removal of the dislocated IOL complex. This unavoidable surgical touch of the iris induced miosis, resulting in a relatively optical pupil size for implantation of the iris-claw IOL [16]. Peripheral iridectomy was not performed. The viscoelastic material was removed, and the sclero-corneal tunnel was subsequently sutured with 10 − 0 nylon. All surgeries were performed by a single retinal specialist (M. K.) with 15 years of experience performing retina surgery. The surgical video is given in additional file 1. Moxifloxacin hydrochloride 0.5% ophthalmic solution (Vigamox, Novartis, Seoul, Korea), prednisolone acetate 1% ophthalmic solution (Pre Forte 1%, Allergan, Seoul, Korea), and bromfenac sodium hydrate 0.1% ophthalmic solution (Bronuck, Taejoon, Seoul, Korea) were administered every hour on the day of surgery, every two hours for the first week following the surgery, and three times a day for the following eight weeks.

### Statistical analysis

A Repeated Measures ANOVA was used to compare ECD, CV, HEX and visual outcomes before and after surgery. Data were analyzed using the SPSS software (version 25.0; IBM Corp., Armonk, NY, USA). The 95% confidence interval was calculated, as appropriate. Statistical significance was set at *P* < 0.05.

## Results

Nine patients (9 eyes) were included in our analysis. Demographic and baseline characteristics of the patients are shown in Table 1. The age (mean ± SD) was 64.89 ± 7.15 years (range 57–76 years), and the mean follow-up duration was 37.93 ± 23.72 months (range 18.07–89.07 months). All the included patients were male. No patients had pseudoexfoliation. The initial mean UDVA was 1.09 ± 0.75, and the initial mean BCVA was logMAR 0.09 ± 0.11; pre-operative ECD was 708.1 ± 150.9 cells/mm^2^.

**Table 1:** Demographic and baseline characteristics of patients who underwent IOL implantation due to IOL dislocation and whose pre-operative ECD was less than 1000 cells/mm^2^ (*n* = 9 patients)

Clinical characteristics	Data
Age, years	64.89 ± 7.15 (median: 62, range 57–76)
Follow-up duration (months)	37.93 ± 23.72 (median: 29, range 18.07–89.07)
Sex, number (%)	
Male	9 (100.0)
Hypertension, no. (%)	4 (44.4)
Diabetes, no. (%)	1 (11.1)
Visual symptoms, no. (%)	9 (100.0)
UDVA, logMAR at initial (Snellen equivalent)	1.09 ± 0.75 (20/250)
BCVA, logMAR at initial (Snellen equivalent)	0.09 ± 0.11 (20/25)
Intraocular pressure (mmHg)	
Pre-operation	16.3 ± 5.7 (9–29)
Post-operation (last follow-up)	14.9 ± 5.7 (9–25)
Pre-operative ECD (cells/mm^2^)	708.1 ± 150.9 (468–941)
Axial length (mm)	24.09 ± 0.95 (22.48–25.28)
Average operation time (minutes)	57.0 ± 12.54 (41–73)
Age, years	64.89 ± 7.15 (median: 62, range 57–76)

Abbreviations: logMAR, logarithm of the minimum angle of resolution; UDVA, uncorrected distance visual acuity; BCVA, best-corrected visual acuity; ECD, endothelial cell density

Age, visual acuity, and intraocular pressure are displayed as mean ± SD

Table 2 shows previous operation history, the cause for IOL dislocation, and corneal endothelial function data at the initial and last follow-up for all nine patients. There was only one patient whose pre-operative ECD was < 500 mm/cm^2^ (Patient 9, ECD = 468 mm/cm^2^). However, his ECD at the last follow-up was 539 mm/cm^2^. Only Patient 7 had an ECD < 500 mm/cm^2^ at the last follow-up; his initial ECD was 518 mm/cm^2^ and the last ECD was 421 mm/cm^2^. No patients developed bullous keratopathy during follow-up. Comparing the ECD values between the initial assessment and all post-operative time points revealed a slight decrease; however, this decline was not statistically significant (*P* = 0.944), as indicated in Table 3.

**Table 2:** Corneal endothelial function data of 9 patients at the initial and last follow-up (*n* = 9)

Patient	Previous operation history	Cause for IOL dislocation	ECD, initial (cells/mm^2^)	ECD, last follow-up (cells/mm^2^)	CT, initial (μm)	CT, last follow-up (μm)	CV, initial (%)	CV, last follow-up (%)	HEX, initial (%)	HEX, last follow-up (%)	Follow-up duration (months)
1	ECCE c PCIOL	Trauma	718	787	577	580	23	39	80	46	29.00
2	ECCE c PCIOL	Unknown	941	703	607	568	37	32	57	54	62.30
3	Phaco c PCIOL	Trauma	686	663	589	589	33	28	54	60	18.20
4	Phaco c PCIOL in sulcus due to PCR	Unknown	662	736	516	554	77	59	47	33	89.07
5	ECCE c PCIOL	Unknown	791	747	682	671	24	29	52	45	18.07
6	ECCE c PCIOL	Unknown	864	611	483	504	40	36	52	57	22.97
7	Phaco c PCIOL in sulcus due to PCR	Unknown	518	421	639	644	43	59	47	41	34.03
8	Phaco c PCIOL	Trauma	725	672	583	593	30	16	63	63	25.00
9	Phaco c PCIOL	Unknown	468	539	534	545	33	32	55	46	42.77
Mean			708.1	653.2	578.9	583.1	37.8	36.7	56.3	49.4	37.93

Abbreviations: ECCE, extracapsular cataract extraction; PCIOL, posterior chamber intraocular lens; Phaco, phacoemulsification; PCR, posterior capsular rupture; IOL, intraocular lens; ECD, endothelial cell density; CT, corneal thickness; CV, coefficient variation of cell area; HEX, percentage of hexagonal cells

**Table 3:** Surgical outcomes after retropupillary iris claw intraocular lens: corneal endothelium function (*n* = 9 patients)

Post-operative period	ECD (cells/mm^2^)	CT (μm3)	CV (%)	HEX (%)
Initial	708.1 ± 150.9	578.9 ± 61.6	37.8 ± 16.1	56.3 ± 10.1
1-month post-operation	656.8 ± 150.1	615.9 ± 59.5	36.1 ± 6.0	51.8 ± 23.7
6-months post-operation	661.8 ± 203.3	588.1 ± 58.0	29.9 ± 8.4	50.5 ± 13.7
1-year post-operation	651.3 ± 104.7	567.3 ± 58.4	27.9 ± 13.8	52.4 ± 17.7
Last follow-up	653.2 ± 114.7	583.1 ± 50.4	36.7 ± 14.2	49.4 ± 9.7
*P*-value	0.944	0.778	0.445	0.443

Abbreviations: ECD, endothelial cell density; CT, corneal thickness; CV, coefficient variation of cell area; HEX, percentage of hexagonal cells

Values are displayed as mean ± SD

A Repeated Measures ANOVA. A P-value less than 0.05 is considered statistically significant

The evaluation of endothelial function after the surgery included a comparison of CT, CV and HEX. The pre-operative and post-operative CT values were 578.9μm and 583.1μm, respectively. The CV values were 37.8% preoperatively and 36.7% postoperatively, whereas the HEX values were 56.3% preoperatively and 49.4%, as detailed in Table 2. Importantly, these measurements demonstrated no noteworthy decrease (*P* = 0.778 for CT, *P* = 0.445 for CV and *P* = 0.443 for HEX), as presented in Table 3.

Immediately after the operation, all patients showed a significant improvement in the UDVA. A post-hoc test showed that there was a statistically significant difference in visual acuity between the initial measurement and all subsequent measurements taken after operation (repeated Measures ANOVA, *p* = 0.004; all relevant pairwise comparisons *p* < 0.004, 0.005, 0.006, and 0.006). Vision was maintained over the follow-up period in all patients (Table 4). The comparison of BCVA between the initial and final assessments did not reveal any significant differences (*P* = 0.896) (Table 4). This result suggests that these patients have achieved their maximum visual potential.

**Table 4:** Surgical outcomes after retropupillary iris claw intraocular lens: visual acuity (*n* = 9 patients)

Post-operative period	UDVA, logMAR (Snellen)	BCVA, logMAR (Snellen)
Initial	1.09 ± 0.75(20/250)	0.09 ± 0.11 (20/25)
1-month post-operation	0.17 ± 0.17(20/29)	0.09 ± 0.11 (20/25)
6-months post-operation	0.15 ± 0.17(20/29)	0.14 ± 0.18 (20/28)
1-year post-operation	0.19 ± 0.11(20/31)	0.19 ± 0.11(20/31)
Last follow-up	0.13 ± 0.15(20/27)	0.13 ± 0.18 (20/27)
*P*-value	0.004	0.896

Abbreviations: UDVA, uncorrected distance visual acuity; logMAR, logarithm of the minimum angle of resolution; BCVA, best corrected visual acuity

Values are displayed as mean ± SD

A Repeated Measures ANOVA. P-value less than 0.05 is considered statistically significant

**Fig. 1:**
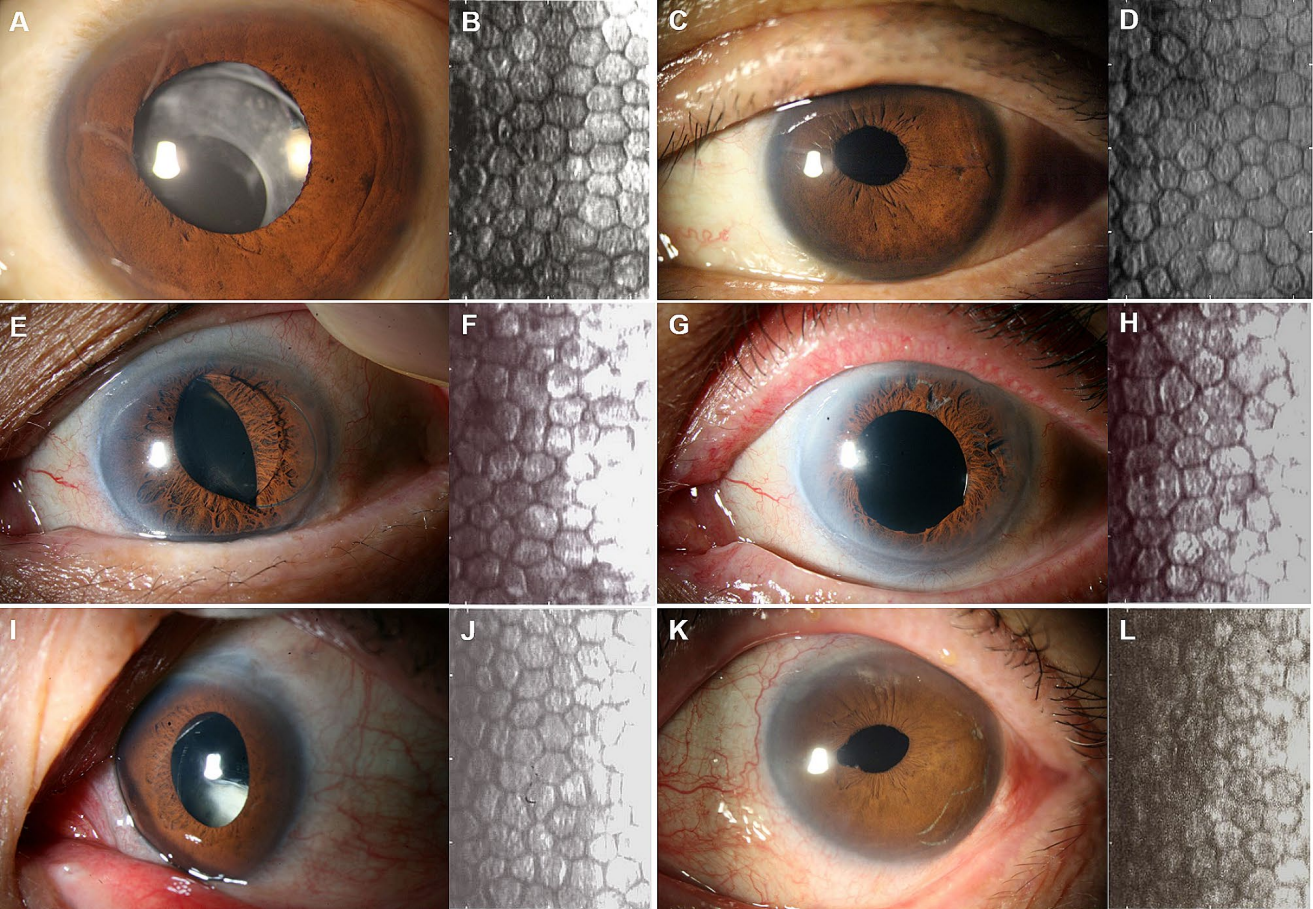
Three representative pre- and post-operative slit photos with ECD specular images. Patient 3 had slight IOL dislocation inferiorly **(A)**, and the pre-operative ECD was 686 mm/cm^2^**(B)**. At last follow-up, the IOL was well-situated **(C)**, and the ECD was 663 mm/cm^2^**(D)**. Patient 6 had inferior IOL haptic dislocated anteriorly **(E)**, and the pre-operative ECD was 864 mm/cm^2^**(F)**. At last follow-up, the IOL was well-situated **(G)**, and the ECD was 611 mm/cm^2^**(H)**. Patient 8 had incomplete IOL dislocation inferiorly **(I)**, and the pre-operative ECD was 725 mm/cm^2^**(J)**. At last follow-up, the IOL was well-situated **(K)**, and the ECD was 672 mm/cm^2^**(L)**. No bullous keratopathy developed in any patient

No intra-operative complications occurred in any of the patients. Seven of the nine patients (77.8%) did not require additional treatment. However, disenclavation of single-haptics occurred in two patients (22.2%; Patients 4 and 9). This complication occurred at 3 years and 8 months post-operatively in patient 4, and at 3 years and 3 months post-operatively in patient 9. Both the disenclaved lenses were re-enclavated without explantation of the originally implanted retropupillary lens. Single-haptic disenclavation recurred a year after the first re-enclavation in Patient 4. The disenclavated haptic was simply re-enclavated, and at the last follow-up 3 years after the second operation, there was no recurrence (Fig. 1).

## Discussion

In the present study, we investigated the safety and visual outcomes of R-IOL implantation in patients with IOL dislocation whose pre-operative ECD was < 1000 cells/mm^2^. There was only one patient in whom the ECD at the last follow-up was < 500 cells/mm^2^; however, none of the patients experienced endothelial decompensation-related complications during the follow-up period. There was significant improvement in the mean UDVA at the last follow-up (average 0.13 logMAR, 20/27 Snellen) compared to the pre-operative mean UDVA (average 1.09 logMAR, 20/250 Snellen).

ECD loss is a common complication of IOL dislocation surgery, and a recent study by Dalby et al. reported ECD loss varying from 11 to 21% [17]. There are a few studies regarding ECD loss after R-IOL implantation. Gonnermann et al. reported an ECD decrease of 5.5% after a mean follow-up of 34 months [12], whereas Durmus et al. reported an ECD loss of 7.2% at a mean follow-up of 9 months [18]. Forlini et al. showed that the post-operative ECD was not statistically significant compared to the pre-operative ECD [13]. Choi et al. reported an ECD decrease of 16% at 1-month post-operatively; however, it remained stable thereafter during the mean follow-up of 38.2 months [14]. The possible cause of ECD loss after R-IOL implantation is suggested to be surgical stress caused by IOL removal through the scleral tunnel [14, 19]. In this study, the ECD loss (7.75% with a mean follow-up of 37.93 months) was similar to that reported in previous studies [12, 14, 17, 18]. The reproducibility of ECD measurements is expected to be in the range of 0–23% [20], and in our study, all but two patients (Patients 2 and 6) had an ECD percentage difference in this range. However, there was no significant difference in the ECD after R-IOL implantation compared with that before R-IOL implantation in this study.

We postulate that the preservation of ECD in these already compromised corneas with extremely low ECD was because additional intra-operative and post-operative measures were taken to minimize the potential damage to the cornea. Intra-operatively, dispersive viscoelastics were used sufficiently and repeatedly during the entire surgical procedure to protect the corneal endothelium. In addition, when the IOL was rescued from the posterior vitreous cavity into the anterior chamber, manipulation of the IOL for removal within the anterior chamber was minimized and extra measures were taken to ensure that the IOL did not come in close contact with the corneal endothelium. Furthermore, IOL removal through the scleral tunnel may cause surgical stress to the corneal endothelium.

In recent times, the sutureless intrascleral fixation technique has gained substantial prominence as a method for surgically fixing IOLs in cases where there is insufficient support from the lens capsule. Notably, Agarwal et al. introduced the glued IOL technique, while Yamane et al. introduced the flanged intrascleral fixation technique, both employing foldable 3-piece IOLs inserted through a-2.8 mm clear corneal incision. These techniques result in less astigmatism, as they necessitate smaller corneal incisions compared to R-IOL, which requires a larger 5.5-mm corneoscleral incision. Nevertheless, the glued IOL technique and Yamane technique require the manipulation of haptics, as well as precise and symmetrical parallel limbus scleral tunnels for correct IOL positioning [21, 22]. The study by Guerin et al. compared the Yamane technique and R-IOL implantation in terms of safety and efficacy. Both techniques resulted in significant improvement in BCVA, and the complication rates were comparable overall. However, the postoperative hyperopic shift was more pronounced in the Yamane group. Although the R-IOL implantation technique depends on the iris condition, it has an advantage over the Yamane technique due to the simplicity of the surgical procedure and less refractive prediction error [23].

A recent advancement comes in the form of the single-piece sutureless scleral fixation IOL (Carlevale, Soleko), designed to be suspended into the posterior chamber through two transscleral plugs. This foldable, one-piece acrylic lens eliminates the need for haptic manipulation. However, ensuring a straight and symmetrical scleral incision is imperative for proper positioning of the plugs [22].

The retropupillary iris claw IOL lens bypasses the need for scleral pockets by directly enclaving onto the iris. However, this technique necessitates an intact iris structure and a larger corneoscleral incision, which could induce astigmatism.

Since a 5.5-mm corneoscleral incision is required for retropupillary iris claw lens im-plantation, we could take full advantage of this sufficiently large incision for one-step smooth removal of the dislocated IOL through the incision, without having to perform any further manipulation within the anterior chamber; such manipulations, including cutting of the IOL, may cause further damage to the corneal endothelium. It was assumed that creating a 5.5-mm corneoscleral incision may likely cause significant damage to the al-ready compromised cornea; however, our study results showed otherwise.

Compared with other suture fixation techniques, our one-step enclavation technique [24] minimizes the surgical time and allows enclavation of the IOL in a matter of seconds. In addition, the post-operative application of any potentially toxic medications to the corneal endothelium was avoided.

In addition to ECD loss, common complications after R-IOL implantation include changes in the IOP, cystoid macular edema, disenclavation of the lens, iris atrophy, and decentration of the lens [25]. Among these complications, only disenclavation of a single haptic occurred in two patients (22.2%). Previous studies reported haptic disenclavation rates of up to 37% [13, 25–27]. Kim et al. reported that if the operation is performed by an inexperienced surgeon, they are more likely to perform insufficient or incorrect haptic enclavation and eventually increase the risk of disenclavation of the haptics [28]. However, in this study, all surgeries were performed by a highly experienced surgeon. Because the number of patients in this study was small, the incidence rate of this complication may seem to be high.

The Artisan aphakic IOL is available in powers ranging from + 2.0 D to + 30.0 D. In this study, we utilized a biconvex model with powers ranging from + 14.5 D to + 20.0 D. Although some researchers posit that peripheral iridectomy is necessary for retropupillary implantation of biconvex IOLs [27], previous studies employing the same Artisan aphakia model for retropupillary implantation did not perform peripheral iridectomy and reported no instances of pupillary block [13, 26, 29]. Folini et al. mentioned that the positioning of the lens posterior to the iris situates the IOL below the iris plane, potentially providing sufficient clearance between the lens optic and the posterior iris surface [13]. One patient experienced elevated IOP at final visit (25mmHg); however, the patient was using glaucoma eyedrops due to a known history of glaucoma to manage increased IOP. Other patients had a normal IOP range after the surgery and during follow-up period.

This study had a few limitations. First, because this study was conducted retrospectively, there were some missing data and the definitive superiority of the treatment could not be evaluated. Second, the sample size was relatively small. A prospective multicentre study with a larger sample size is required to confirm our findings.

In conclusion, R-IOL implantation did not result in a statistically significant decline in corneal endothelial function in patients with preoperatively low ECD, and it significantly improved the mean UDVA postoperatively. Our findings suggest that R-IOL implantation in patients with extremely low ECD (< 1000 cells/mm^2^) is a safe and effective option for treating IOL dislocation. However, long-term follow-up is necessary to confirm our findings.

## Data Availability

The datasets generated during and/or analyzed during the current study are available from the corresponding author on reasonable request.
